# Distinguishing Fast and Slow Processes in Accuracy - Response Time Data

**DOI:** 10.1371/journal.pone.0155149

**Published:** 2016-05-11

**Authors:** Frederik Coomans, Abe Hofman, Matthieu Brinkhuis, Han L. J. van der Maas, Gunter Maris

**Affiliations:** 1 Psychological Methods, University of Amsterdam, Amsterdam, Netherlands; 2 Quantitative Psychology and Individual Differences, KU Leuven, Leuven, Belgium; 3 Cito Institute for Educational Measurement, Arnhem, Netherlands; Mälardalen University, SWEDEN

## Abstract

We investigate the relation between speed and accuracy within problem solving in its simplest non-trivial form. We consider tests with only two items and code the item responses in two binary variables: one indicating the response accuracy, and one indicating the response speed. Despite being a very basic setup, it enables us to study item pairs stemming from a broad range of domains such as basic arithmetic, first language learning, intelligence-related problems, and chess, with large numbers of observations for every pair of problems under consideration. We carry out a survey over a large number of such item pairs and compare three types of psychometric accuracy-response time models present in the literature: two ‘one-process’ models, the first of which models accuracy and response time as conditionally independent and the second of which models accuracy and response time as conditionally dependent, and a ‘two-process’ model which models accuracy contingent on response time. We find that the data clearly violates the restrictions imposed by both one-process models and requires additional complexity which is parsimoniously provided by the two-process model. We supplement our survey with an analysis of the erroneous responses for an example item pair and demonstrate that there are very significant differences between the types of errors in fast and slow responses.

## Introduction

Modeling the relationship between response time and accuracy in problem solving is a daunting task. However, the advent of computerized testing data becoming available on a large scale allows for a detailed study of the interplay between speed and accuracy. We consider the problem in its simplest non-trivial form. That is, we confine our attention to the situation where persons try to solve two problems only; their response time is coded as either fast or slow, and we only register whether or not the response is correct. Although simplistic, our setting gives us access to data from a large number of item pairs, spanning such diverse subject areas as basic arithmetic, language learning, and intelligence-related problems, with large numbers of independent observations per item pair.

As response time is coded as a binary variable, the response of a person to a single item can be represented with two binary variables, *x*_*i*_ and *y*_*i*_, as follows:

$$\begin{array}{c} x_{i}=\left\{\begin{array}{cc} 1 & \text{if}\;\text{the}\;\text{person}\;\text{solves}\;\text{item}\;i\;\text{correctly}\;,\\ 0 & \text{otherwise} \end{array}\right. \end{array}$$ (1)

and

$$\begin{array}{c} y_{i}=\left\{\begin{array}{cc} 1 & \text{if}\;\text{the}\;\text{person}\;\text{solves}\;\text{item}\;i\;\text{fast}\;,\\ 0 & \text{otherwise}\;. \end{array}\right. \end{array}$$ (2)

Thus, there are four possible ways to answer a single item: fast and incorrectly (*x*_*i*_ = 0, *y*_*i*_ = 1); slowly and incorrectly (*x*_*i*_ = 0, *y*_*i*_ = 0); slowly and correctly (*x*_*i*_ = 1, *y*_*i*_ = 0); and fast and correctly (*x*_*i*_ = 1, *y*_*i*_ = 1). Therefore, there are 16 possible ways to answer an item pair. The type of items we consider are open-ended problems that are administered with the same time limit applying to each of the problems. We choose, quite arbitrarily, to define fast responses as those responses that are given before half of the time has expired and to call all other responses slow responses. Although arbitrary, this choice suffices to show how many models for response time and accuracy fail to explain the observed relationships and points the way to the kind of model that could successfully explain them.

As an example, we discuss the item pair that comprises the following two multiplication problems: 100 × 3000 (item 1) and 80 × 2 (item 2). The answer patterns of 18744 subjects that answered this pair of items within one day are summarized in the contingency table displayed in Table 1. All observations, i.e. all response pairs (*x*_1_, *y*_1_; *x*_2_, *y*_2_), correspond to different subjects, which guarantees that the observations are independent. These data are obtained from Math Garden, a computerized adaptive practice environment in which children can practice their mathematical abilities [1]. This framework and the way the data are extracted from it is discussed in more detail in the first Methods subsection. The layout of Table 1 is in line with what one expects when fast (in)correct responses reflect a (lower) higher proficiency than do slow (in)correct responses, but for all purposes the layout is insignificant. Specifically, we infer higher proficiency as we move from left to right, from top to bottom, and from the north-west corner to the south-east corner of the table.

**Table 1:** Item pair contingency table for items 1 (100 × 3000) and 2 (80 × 2) constructed from 18744 response pairs.

	(*x*_2_ = 0, *y*_2_ = 1)	(*x*_2_ = 0, *y*_2_ = 0)	(*x*_2_ = 1, *y*_2_ = 0)	(*x*_2_ = 1, *y*_2_ = 1)
(*x*_1_ = 0, *y*_1_ = 1)	435 (1)	245 (2)	428 (3)	1668 (4)
(*x*_1_ = 0, *y*_1_ = 0)	256 (5)	229 (6)	487 (7)	1108 (8)
(*x*_1_ = 1, *y*_1_ = 0)	509 (9)	586 (10)	1382 (11)	2245 (12)
(*x*_1_ = 1, *y*_1_ = 1)	1227 (13)	786 (14)	1624 (15)	5529 (16)

The numbers between parentheses indicate the enumeration that is used throughout the text to indicate the events in the item pair contingency table. The cells 1, 4, 13, and 16 constitute the events for which both responses on the item pair are fast (fast-fast responses). The cells 6, 7, 10, and 11 constitute the events for which both responses on the item pair are slow (slow-slow responses). All remaining cells constitute the events for which the speed of both responses on the item pair differs.

In the psychometric literature, a range of models can be found that relate both response time and accuracy to person as well as item characteristics. All these models are based on the standard psychometric assumption of local independence: the responses (*x*_*i*_, *y*_*i*_) and (*x*_*j*_, *y*_*j*_) of *a single person* on *two distinct items i* and *j* are conditionally independent given a set of latent parameters O. This means that this set of parameters completely explains how the responses are correlated:

$$\begin{array}{c} P\left(x_{i},y_{i},x_{j},y_{j}|O\right)=P\left(x_{i},y_{i}|O\right)P\left(x_{j},y_{j}|O\right)\;. \end{array}$$ (3)

Contingency tables such as the one displayed in Table 1 contain the responses of many persons such that we can only observe the manifest probability distribution

$$\begin{array}{c} P\left(x_{i},y_{i},x_{j},y_{j}\right)=\int P\left(x_{i},y_{i}|O\right)P\left(x_{j},y_{j}|O\right)f\left(O\right)\text{d}O\;, \end{array}$$ (4)

where f(O) denotes the joint distribution of the latent parameters in O in the population. Different types of latent structures result in different manifest distributions. Hence, despite the fact that we cannot directly observe the latent structure, it is possible to draw some conclusions about this structure by simply looking at the cumulative data in a contingency table. By dichotomizing response times as done in Eq (2) we enormously reduce the complexity of the models that are eligible to describe the data. However, even in this much simplified form, the manifest probabilities defined in Eq (4) still enable us to distinguish between three types of psychometric accuracy-response time models available in the literature. These types of models are distinguished based on how the correlation between accuracy *x*_*i*_ and response time *y*_*i*_ of *a single person* on *a single item* *i* is modeled:

1. **Models in which *x*_*i*_ and *y*_*i*_ are conditionally independent**. In these models *x*_*i*_ and *y*_*i*_ are conditionally independent given the set of parameters O:
  $$\begin{array}{c} P\left(x_{i},y_{i}|O\right)=P\left(x_{i}|O\right)P\left(y_{i}|O\right)\;. \end{array}$$ (5)
  Generically these models have two types of parameters: one type that governs the response time (the ‘speed’) and one type that governs the accuracy (the ‘ability’). Correlations are introduced only at the level of an external model, i.e. at the level of f(O), that characterizes the distribution in the population of the latent parameters in O. Accuracy and response time are correlated in the population only because the underlying latent parameters, speed and ability, are correlated in the population. The correlation between accuracy and response time is thus spurious and disappears when both latent parameters are kept fixed. Conditionally independent models are captured by van der Linden’s hierarchical framework [2]. From now on we will indicate this conditionally independent type of model with CIM.
2. **Models in which *x*_*i*_ and *y*_*i*_ are conditionally dependent**. For these models, $P(x_{i},y_{i}|O)$ cannot be factorized as in Eq (5). This means that the correlations between *x*_*i*_ and *y*_*i*_ are structural and cannot be ‘explained away’ by additional latent parameters. In more technical terms, *x*_*i*_ and *y*_*i*_ are coupled in the sufficient statistics for the model parameters, i.e. the model contains explicit interaction terms:
  $$\begin{array}{c} P\left(x_{i},y_{i}|O\right)∼\text{exp}\left(x_{i}y_{i}O\right)\;. \end{array}$$ (6)
  These models have only one type of parameter that governs both the speed and the accuracy. A prototype example of this type of model is the Signed Residual Time (SRT) scoring rule model of [3]. In this model, the parameter that governs the probability to answer fast is the absolute value of the parameter that governs the probability to answer correctly. From now on we will indicate this conditionally dependent type of model with CDM.
3. **Models in which *x*_*i*_ is contingent on *y*_*i*_**. A third way to model the correlation between *x*_*i*_ and *y*_*i*_ is by assuming that (*x*_*i*_|*y*_*i*_ = 1) is governed by a different parameter than (*x*_*i*_|*y*_*i*_ = 0). This gives rise to a two-level branching model that explicitly distinguishes between fast and slow responses. It has three types of parameters: one that governs the accuracy for fast responses, one that governs the accuracy for slow responses and one that governs the mixing of fast and slow responses. This two-process model was first introduced in [4] and is a specific example of a multinomial process tree model [5]. Since the two-level branching model is saturated on the contingency table two different truncations of this two-process model, labeled the 2P&3I truncation and the 3P&2I truncation, are used in the analyses. The 2P&3I truncation is obtained from the two-level branching model by constraining the person parameters that govern the accuracies for fast and slow responses to be equal such that the truncated model only has two person parameters in addition to the three item parameters. The 3P&2I truncation is obtained from the two-level branching model by constraining the item parameters that govern the accuracies for fast and slow responses to be equal.

It is important to stress that these three model types are distinguished solely on the basis of the different latent mechanisms they employ to produce the observed correlations between speed and accuracy in the population. We do not make any assertions about the within-person processes that lie at the basis of these latent structures.

All of these model types and their mutual relations will be discussed in more detail in the Results and Methods section. We end this Introduction by giving a flavor of the type of analysis that is used in the Results section: we compare all three model types on the basis of the observations of the example item pair displayed in Table 1. Based on the empirical data in Table 1, we computed the estimated frequencies of the 16 answer patterns using the CIM, CDM and 2P&3I and 3P&2I truncations of the two-level branching model. The estimated frequencies of these models are displayed in Table 2. The corresponding Pearson goodness-of-fit *χ*^2^-statistics have values 365.24 (CIM), 1139.86 (CDM), 0.20 (2P&3I) and 47.19 (3P&2I). The data for the example item pair clearly violates the restrictions imposed by both the CIM and the CDM on the contingency table probabilities. Both two-level branching model truncations give a much better description of the data than the CIM and CDM do. In the next section we demonstrate that the conclusions reached for this example item pair generalize by discussing the results of a survey in which we analyzed numerous different item pairs from different domains.

**Table 2:** Estimated frequencies computed from CIM, CDM, 2P&3I and 3P&2I.

	(*x*_2_ = 0, *y*_2_ = 1)		(*x*_2_ = 0, *y*_2_ = 0)		(*x*_2_ = 1, *y*_2_ = 0)		(*x*_2_ = 1, *y*_2_ = 1)	
(*x*_1_ = 0, *y*_1_ = 1)	**435**	435.00	**245**	214.50	**428**	657.99	**1668**	1571.62
		435.00		266.63		439.71		1191.26
		435.00		245.00		423.88		1672.12
		435.00		245.00		428.00		1572.26
(*x*_1_ = 0, *y*_1_ = 0)	**256**	286.50	**229**	229.00	**487**	582.50	**1108**	878.89
		234.37		386.52		1047.15		1235.43
		256.00		229.00		491.12		1103.88
		256.00		229.00		582.74		1108.00
(*x*_1_ = 1, *y*_1_ = 0)	**509**	740.06	**586**	490.50	**1382**	1382.00	**2245**	2212.55
		339.76		920.47		1085.97		2059.04
		513.12		581.88		1382.00		2245.00
		509.00		490.26		1382.00		2245.00
(*x*_1_ = 1, *y*_1_ = 1)	**1227**	1323.38	**786**	554.06	**1624**	1656.45	**5529**	5529.00
		809.12		954.60		1809.96		5529.00
		1222.88		790.12		1624.00		5529.00
		1322.741		786.00		1624.00		5529.00

Estimates are obtained based on the data in Table 1. Every cell contains the observed frequency (in bold) together with 4 expected frequencies corresponding to, respectively from top to bottom, the CIM, the CDM, the 2P&3I truncation and the 3P&2I truncation. The corresponding Pearson goodness-of-fit *χ*^2^-statistics are $\chi_{CIM}^{2}=365.24$ (20.52), $\chi_{CDM}^{2}=1139.86$ (26.12), $\chi_{2P\&3I}^{2}=0.20$ (10.83) and $\chi_{3P\&2I}^{2}=47.19$ (10.83), where the numbers in brackets denote the statistics’ bounds corresponding to *p* = 0.001 for the corresponding degrees of freedom.

## Results

We compared the fit on item pair contingency tables of the CIM and CDM with the fit of a two-level branching model that explicitly distinguishes between fast and slow responses. The relations between the models are discussed in the first subsection of this Results section. In the second subsection we will discuss the results of a survey for which we estimated the CIM, the CDM and two truncations of the two-level branching model on a large amount of item pairs stemming from 4 basic arithmetic domains: multiplication, addition, subtraction and division. In the third subsection we discuss the results of a different survey over item pairs from domains outside basic arithmetic: first language learning, the game of Set and chess. In the fourth subsection we strengthen the support for the two-level branching model by analyzing the incorrect responses in Table 1 and show that there are significant differences between the kind of errors made when the response is fast and those made when the response is slow.

### How the three types of models relate

In this subsection we will discuss the relations between the different model types. We will start with the two-level branching model and discuss two particular truncations of this model, the 2P&3I truncation and the 3P&2I truncation, that preserve the explicit distinction between fast and slow responses. As such these truncations will still be labeled as two-process models. We will also explain how the CIM and CDM can be considered as truncations of the two-level branching model for which the explicit distinction between fast and slow responses disappears. As such they are labeled as one-process models.

**Fig 1:**
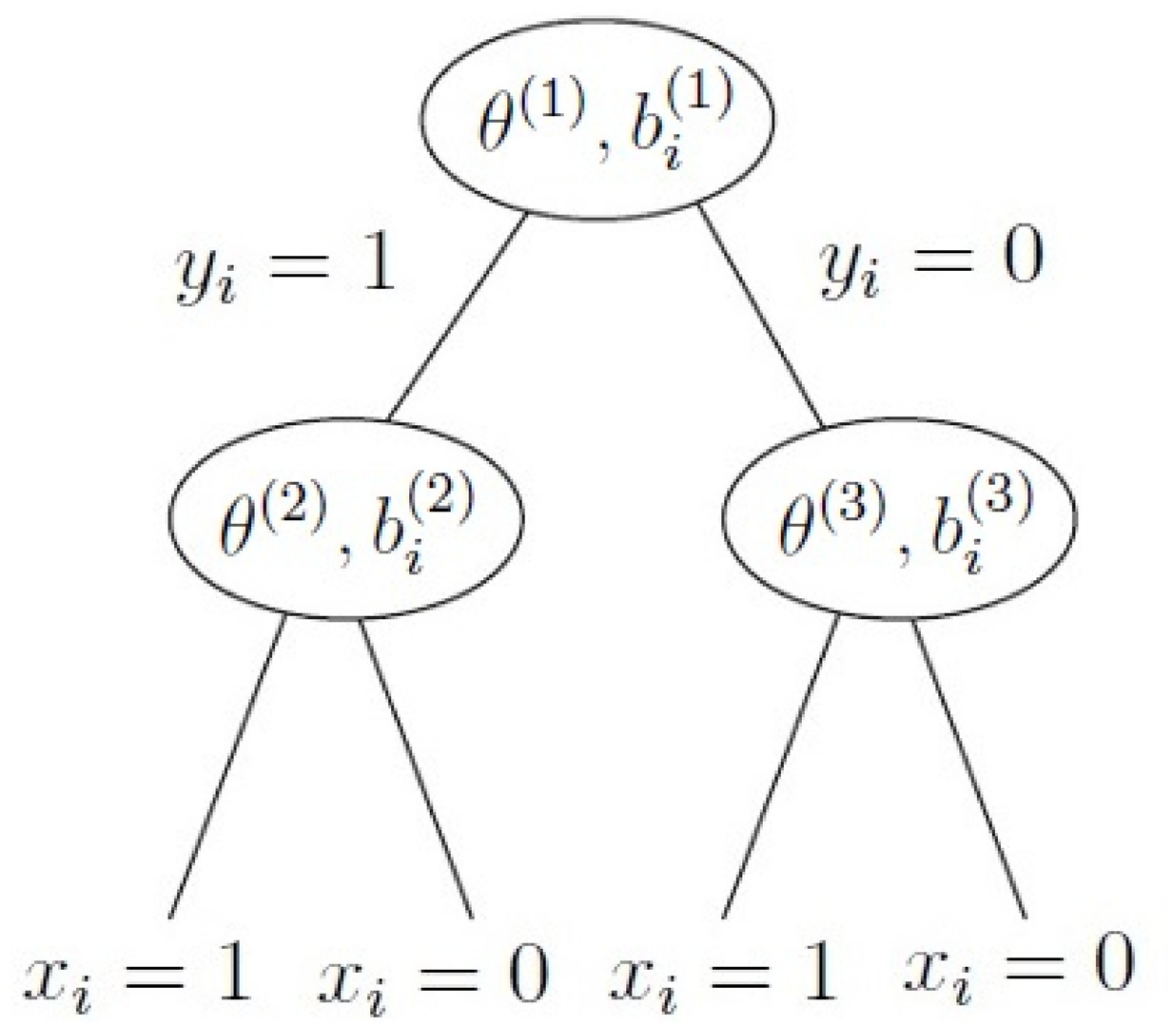
Schematic description of a two-level branching model. The first level distinguishes fast (*y*_*i*_ = 1) and slow (*y*_*i*_ = 0) responses, whereas the second level distinguishes correct (*x*_*i*_ = 1) and incorrect (*x*_*i*_ = 0) responses. In the nodes, the person and item parameters of the corresponding Rasch models are displayed. The left branch of the first node corresponds to the probability of answering fast, the left branch of the second node corresponds to the probability of answering correctly given that the response is fast, and the left branch of the third node corresponds to the probability of answering correctly given that the response is slow.

The two-level branching model of Partchev and De Boeck [4] has first-level branches that differentiate between fast and slow and second-level branches that further differentiate between correct and incorrect. The branching structure is displayed in Fig 1. The probability to go left at node *s* ∈ {1, 2, 3} is given by a Rasch model with parameters specified in the node in Fig 1:

$$\begin{array}{c} P\left(\text{go}\;\text{left}\;\text{at}\;\text{node}\;s|\theta^{\left(s\right)},b_{i}^{\left(s\right)}\right)=\frac{\text{exp}\left(\xi_{i}^{\left(s\right)}\right)}{1+\text{exp}\left(\xi_{i}^{\left(s\right)}\right)}\;, \end{array}$$ (7)

where $\xi_{i}^{\left(s\right)}=\theta^{\left(s\right)}-b_{i}^{\left(s\right)}$. This model explicitly distinguishes fast and slow responses and considers them as arising from two different processes: one governed by *θ*^(2)^ and one governed by *θ*^(3)^. The mixing of both processes is governed by *θ*^(1)^. The model has to be supplemented with a multivariate distribution *f*(*θ*^(1)^, *θ*^(2)^, *θ*^(3)^) that describes the distribution of the three latent parameters in the population.

**Fig 2:**
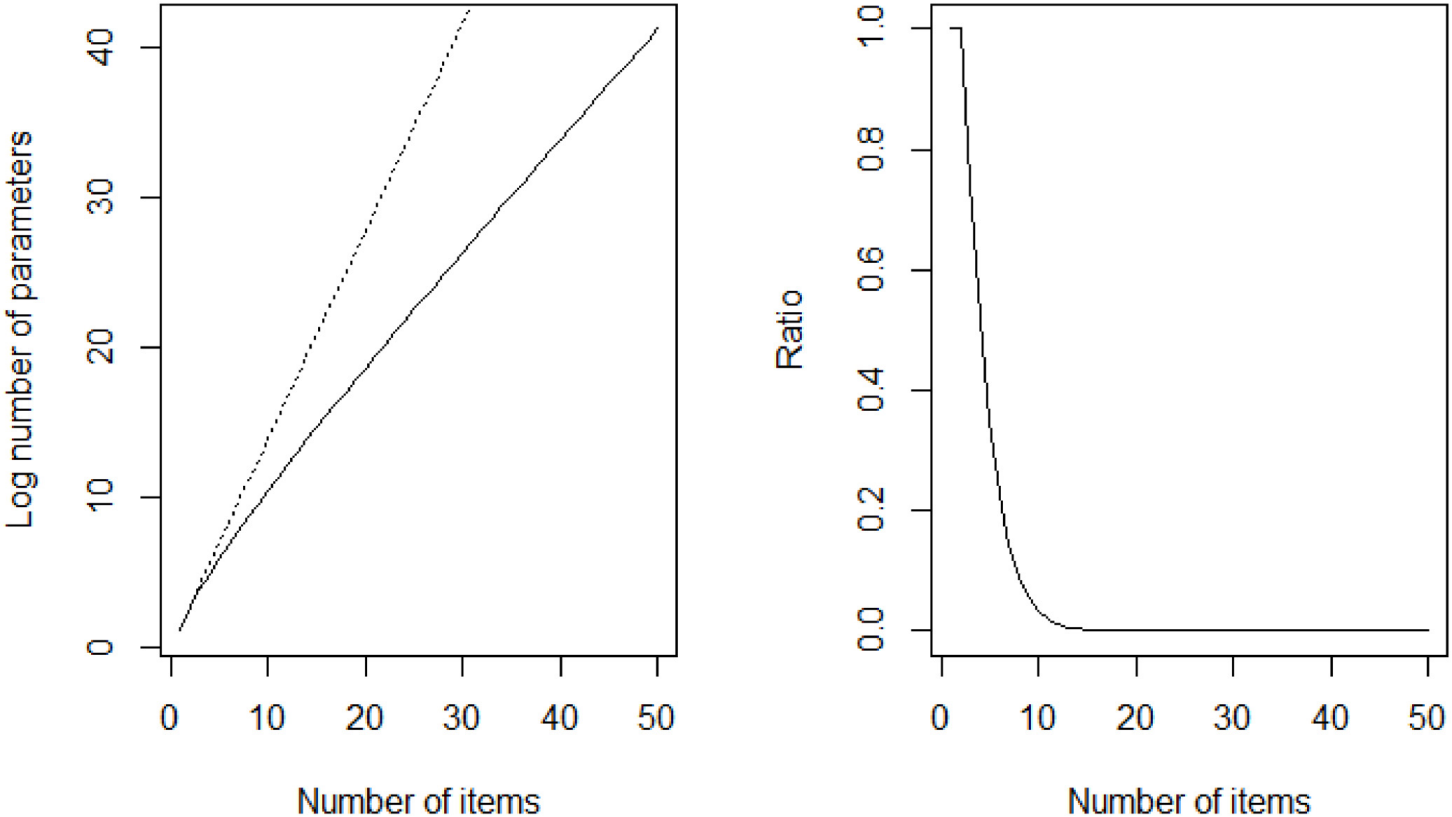
Number of parameters vs. number of items. The left plot compares the log number of parameters of the two-level branching model (solid) and the saturated model (dashed) on an *N*-item contingency table as function of *N*. The right plot displays the ratio of the number of parameters of the two-level branching model over the number of parameters of the saturated model (both on an *N*-item contingency table) as function of *N*.

The restrictions imposed by this model on item pair contingency tables are discussed in detail in the second Methods subsection where it is concluded that this model is saturated on such tables. This means that it has a parameter for every cell in the contingency table and thus will always give a perfect fit. Therefore we will look at parameter truncations of this model that are not saturated on the item pair contingency table. Before considering these truncations, we note that although the model is saturated on the item pair contingency table, we still consider it very parsimonious. This is so because if we consider *N* > 2 items and look at *N*-item contingency tables, the difference between the number of parameters of the saturated model and the number of parameters of the two-level branching model increases exponentially with *N*. This is illustrated in Fig 2.

Partchev and De Boeck [4] consider three parameter truncations of the two-level branching model, two for which the explicit distinction between fast and slow processes is preserved (the 2P&3I truncation and the 3P&2I truncation) and one for which it disappears (the CIM). We will discuss these truncations together with one additional truncation of the latter type (the CDM).

**Truncations that preserve the explicit distinction between fast and slow:**
- The 2P&3I truncation: *θ*^(2)^ = *θ*^(3)^. Here, there is only one ability for fast and slow responses but the item parameters differ.
- The 3P&2I truncation: $b_{i}^{\left(2\right)}=b_{i}^{\left(3\right)}$. Here, there is only one set of difficulties for fast and slow responses but the person parameters differ.

The manifest probabilities, which were generically defined in Eq (4), that correspond to these models are discussed in detail in the second Methods subsection. Both the 2P&3I and 3P&2I truncations have 14 parameters on the item pair contingency tables.

**Truncations that do not preserve the explicit distinction between fast and slow:**
- The CIM: *θ*^(2)^ = *θ*^(3)^ and $b_{i}^{\left(2\right)}=b_{i}^{\left(3\right)}$. Here, there is only one ability and one set of difficulties for fast and slow responses.
- The CDM: *θ*^(2)^ = *θ*^(3)^ and $b_{i}^{\left(2\right)}=b_{i}^{\left(3\right)}$ and $\xi_{i}^{\left(1\right)}=\left|\theta^{\left(2\right)}-b_{i}^{\left(2\right)}\right|$. Here, there is only one ability and one set of difficulties for fast and slow responses. Moreover, the response time is governed by the absolute value of the difference of these parameters $|\theta^{\left(2\right)}-b_{i}^{\left(2\right)}|$.

The manifest probabilities that correspond to these models are discussed in detail in the third and fourth Methods subsection. The CIM has 10 parameters on the item pair contingency tables and the CDM has 7 parameters on the item pair contingency tables.

To end this subsection we mention that for all these types of models we assume that the parameters that govern the latent structures are fixed per observation in the contingency table. That is, we do not allow within-person variability of the latent parameters and assume that both responses that make up an observation were given by a subject with fixed latent parameters. This stationarity assumption, which is standard in psychometric response-time modeling [2, 6], is based on the following arguments:

- Both items are administered under the same framework, the Math Garden framework, which is described in the first Methods subsection.
- To exclude the impact of changing environment variables such as learning effects or the increase of fatigue, we limit the observations to those that correspond to response pairs that were given within the same day. Since most subjects only practice for a very limited time per day, this effectively results in contingency tables where the majority of the observations corresponds to two responses that were given in a time interval of the order of minutes. For example, for the data in Table 1 90% of the subjects answered both items within 10 minutes.
- There is no fixed order in which subjects answered both items. For example, for the data in Table 1 51% of the subjects answered item 1 first.

In the next subsections we will determine the fit of these models on a large number of item pairs stemming from a number of different domains.

### Empirical comparison of the models (1): a survey of item pairs from basic arithmetic domains

As alluded to above, the features observed in Tables 1 and 2 are not limited to the specific item pair that we focused on in the Introduction. We found that these features are persistent over different item pairs stemming from a diverse range of subject areas. We conducted a survey in the Math Garden framework covering a number of basic arithmetic domains—addition, subtraction, multiplication, and division. For each domain, we extracted the item pair contingency tables corresponding to all 435 possible pairs of the 30 most played items in that domain (according to the period from 01-03-2012 to 01-07-2014). We estimated the CIM, the CDM and the 2P&3I and 3P&2I truncations for each of these item pair contingency tables and computed the corresponding *χ*^2^-statistics. These statistics have a *χ*^2^-distribution with the appropriate number of degrees of freedom only if all of the following requirements are met [7]:

- The observations are independent.
- The corresponding contingency table has a sufficiently large number of observations.
- At least 80% of the cells has an expected frequency of at least 5 and none of the cells has an expected frequency below 1.

The first requirement is met in our analysis since all observations in the contingency tables correspond to different subjects. In order to meet the last two requirements we used the following exclusion policy:

- If an item pair has a corresponding contingency table with less than 500 observations it is excluded from the analyses of all 4 models.
- For a given item pair and under a given model, if more than 3 expected cell frequencies do not exceed 5 or at least 1 expected cell frequency does not exceed 1, the item pair is excluded from the analysis of that particular model.

The results of this survey are displayed in Table 3. As can be seen in this table, we find that the majority of the item pairs in all the considered domains provide extensive evidence to reject the CIM and CDM model descriptions and that overall the 2P&3I truncation fits best.

**Table 3:** Survey of violations.

domain	CIM *χ*^2^ > 20.52	CDM *χ*^2^ > 26.12	2P&3I *χ*^2^ > 10.83	3P&2I *χ*^2^ > 10.83
multiplication	**96%** (427)	**100%** (426)	**0%** (432)	**31%** (432)
division	**95%** (346)	**100%** (362)	**1%** (344)	**21%** (345)
addition	**61%** (339)	**100%** (347)	**1%** (343)	**9%** (340)
subtraction	**60%** (435)	**100%** (435)	**0%** (435)	**20%** (435)

For each Math Garden domain indicated in the left column, all 435 possible item pairs of the 30 most played items in the domain are considered. All item pairs with less than 500 administrations are discarded. For each remaining item pair, an item pair contingency table is constructed for which the CIM, CDM, 3P&2I and 2P&3I are estimated and the four corresponding *χ*^2^-test statistics are computed. Under a given model for a specific item pair, if one or more of the expected cell frequencies is below 1 or if more than 3 cell frequencies are below 5, the item pair is excluded from the analysis of that specific model. The total number of remaining item pairs per domain and per model is indicated in parentheses in the corresponding table entry. The boldface number in every table entry indicates what percentage of this number of remaining item pairs has a *χ*^2^-value that exceeds the *p* = 0.001 threshold for the corresponding number of degrees of freedom. This threshold is 20.52 for CIM (p = 0.001 for 15-10 degrees of freedom), 26.12 for CDM (p = 0.001 for 15-7 degrees of freedom), 10.83 for 2P&3I (p = 0.001 for 15-14 degrees of freedom) and 10.83 for 3P&2I (p = 0.001 for 15-14 degrees of freedom).

### Empirical comparison of the models (2): a survey of item pairs from language learning, Set and chess

The simplicity of our setup, which makes use of only two items, allows us to extend it beyond the basic arithmetic domains discussed above and apply it to a diverse number of subject areas for which less data is available. As a demonstration, we add here a number of analyses concerning item pairs from the card game ‘Set’, a language learning game called ‘Letter Chaos’, and chess. We analyse the data by estimating the CIM, the CDM and the 2P&3I and 3P&2I truncations and show that this leads to similar conclusions as drawn in the previous subsection.

The Set game [8] is a card game that has been intensively studied because it is able to elicit a whole range of complex cognitive processes with only a very simple setup [9]. Letter Chaos is a language game, the aim of which is to recognize a word from a sequence of letters that are randomly shuffled. Set is built into the Math Garden framework and Letter Chaos is built into the computerized adaptive practice framework Language Sea, which is the language variant of the Math Garden framework. Both Set and Letter Chaos games have items that are scored with the SRT scoring rule Eq (9) with a 20 second deadline. In the same way as described in the first Methods subsection, we extracted the item pair contingency tables corresponding to all 45 possible pairs of the 10 most played items in the Set and Letter Chaos domains (according to the period from 01-03-2012 to 01-07-2014). We estimated the CIM, the CDM and the 2P&3I and 3P&2I truncations on these tables, and computed the corresponding *χ*^2^-test statistics. The results are summarized in Table 4. We employed the same exclusion policy as described in the previous subsection.

**Table 4:** Survey of violations (2).

domain	CIM *χ*^2^ > 20.52	CDM *χ*^2^ > 26.12	2P&3I *χ*^2^ > 10.83	3P&2I *χ*^2^ > 10.83
Set	**100%** (24)	**100%** (45)	**0%** (22)	**0%** (24)
Letter Chaos	**15%** (39)	**100%** (45)	**0%** (39)	**0%** (39)
Chess	**90%** (348)	**97%** (401)	**0%** (153)	**39%** (227)

For Set and Letter Chaos, all 45 possible item pairs of the 10 most played items are considered. All item pairs with less than 500 administrations are discarded. The analysis of the remaining item pairs in these domains is equivalent to that described below Table 3. For chess, 780 item pairs of 40 items in the Amsterdam Chess Test I are considered. Each item pair has a fixed number of 259 observations and thus we do not employ the ‘500-observations-requirement’ here. The rest of the analysis is equivalent to that described below Table 3. However, it has to be noted that 310 of the 627 item pairs that were excluded from the analysis of the 2P&3I model have sparse contingency tables for which the corresponding set of 2P&3I maximum likelihood equations, given in Eq (16), has no solution.

The chess data are obtained from the Amsterdam Chess Test I (part A) [10]: 259 participants all answered the same 40 items and the accuracies and response times were recorded. Based on this information, we were able to build all 780 corresponding item pair contingency tables. We estimated the CIM, the CDM and the 2P&3I and 3P&2I truncations on these tables and computed the corresponding *χ*^2^-test statistics. The results are summarized in Table 4. We employed the same exclusion policy as described in the previous subsection except for the ‘500-observations-requirement’ since all tables have a fixed number of 259 observations.

It is clear from Table 4 that the majority of the data violate the restrictions imposed by the CIM and CDM and that it agrees much better with the 2P&3I and 3P&2I truncations.

### Collateral evidence: an analysis of fast and slow errors

To provide additional evidence for the two-process explanation of Table 1, we carried out an analysis of the incorrect responses in the table and showed that there are significant differences between the most common fast errors and the most common slow errors. We separately analyzed the 4856 incorrect responses for item 1 and the 4273 incorrect responses for item 2 and focused on the 10 most common incorrect responses for each item. These responses are displayed in Table 5, together with their corresponding frequencies. These frequencies are split up according to whether the response is fast or slow.

**Table 5:** Error contingency table.

item 1 errors	fast	slow	item 2 errors	fast	slow
*30000*	1326	717	*1600*	366	192
*100000*	458	287	*140*	208	174
*3000000*	299	249	*120*	203	87
*3000*	133	82	*800*	186	95
*400000*	50	126	*40*	154	40
*1300000*	17	66	*802*	101	89
*4000*	45	32	*180*	95	75
*10000*	49	26	*600*	65	103
*30*	42	14	*16*	86	60
*9000*	18	36	*200*	80	50
residual	339	445	residual	883	881

For items 100 × 3000 (item 1) and 80 × 2 (item 2) the 10 most common incorrect responses are displayed together with their corresponding frequencies and are split up based on the speed of the response. This data is obtained from the Math Garden framework for the period from 01-03-2012 to 01-07-2014. Note that we left out the response *0* from the analysis because it was set as the default answer in the Math Garden framework for some time. Note that the sum of the frequencies of the fast and slow errors of, respectively, the first (second) item equal the sum of the first and second rows (columns) in Table 1, as they should.

Applying a *χ*^2^-test to both tables leads to the following test statistics (the *χ*^2^-threshold for a *p* = 0.001 significance level and 10 degrees of freedom is 29.59):

$$\begin{array}{c} \text{item}\;\text{1:}\;\chi^{2}=248.11\;,\;\text{item}\;\text{2:}\;\chi^{2}=147.13\;, \end{array}$$ (8)

indicating that there are very significant differences between the types of fast errors and the types of slow errors on this particular item pair.

## Discussion

The results discussed in the previous section clearly demonstrate that the standard psychometric one-process models, the CIM and CDM, are not consistent with the data. Table 3 summarizes the results for the basic arithmetic pairs under consideration. For the CDM each of the item pairs that were analyzed provides enough evidence to reject that model at the *p* = 0.001 level. The evidence for rejecting the CIM at the *p* = 0.001 level is slightly more nuanced but still very convincing. Similar conclusions can be drawn from the Letter Chaos, Set and chess data, the analysis of which is summarized in Table 4. Overall, we conclude that the observed frequency distributions on item pair contingency tables extracted from this diverse range of domains require more complexity than the simple CIM and CDM one-process models can offer.

The two-level branching model introduced in Eq (7) and Fig 1 introduces extra complexity in a very parsimonious manner. The model can be considered parsimonious since the difference of the number of parameters of the saturated model and the two-level branching model on an *N*-item contingency table increases exponentially with *N* as can be seen in Fig 2. We focused on two particular truncations of the two-level branching model, the 2P&3I truncation and the 3P&2I truncation, that preserve the explicit distinction between fast and slow responses and thus can still be labeled two-process models. Table 3 shows that the vast majority of the basic arithmetic item pairs that we investigated agree with the 2P&3I truncation. On the Letter Chaos, Set and chess data the model performs equally well: all of the item pairs in these domains that we investigated agree with the 2P&3I model. It has to be noted that the number of chess item pairs analyzed under this model is much lower than the number of chess item pairs that were analyzed under the other models. As mentioned already under the Table 4 this is mainly due to the fact that many of the chess item pair contingency tables contain many cells with frequency zero which causes the corresponding system of maximum likelihood Eq (16) to have no solution. The 3P&2I truncation does not agree as well with the data as the 2P&3I model does. Table 3 shows that substantial fractions of the analyzed basic arithmetic item pairs reject the model at the *p* = 0.001 level. As can be seen from Table 4 the model performs much better on Letter Chaos and Set item pairs but again performs less good in the chess domain. Additional evidence for an explicit distinction between fast and slow responses is given by our analysis of fast and slow errors, which is displayed in Table 5 and which indicates that there is a significant difference between the types of slow and fast errors.

Our results are in agreement with the findings of [4]. There, the authors investigated the performance of the two-level branching model, the 2P&3I truncation, the 3P&2I truncation and the 2P&2I truncation (which is equivalent with our CIM) on data from a Raven-like matrices test and a verbal analogies test. They concluded that the full model fits best but that the abilities governing the fast and slow accuracies (*θ*^(2)^ and *θ*^(3)^) are rather strongly correlated. In our case, the full model is saturated on the item pair contingency table but we find that the model in which *θ*^(2)^ = *θ*^(3)^ perfectly agrees with the data. The question remains if this strict equality survives when *N*-item contingency tables with *N* > 2 are considered. However, it is hard to find such contingency tables that are non-sparse since the adaptivity of the Math Garden framework makes that the sparsity of these tables quickly increases with *N*.

The findings discussed above seem to fit into the broader research framework of dual-process modeling. Dual-process theories assume that there are two qualitatively different modes of processing that underlie such ‘higher order’ cognitive phenomena as reasoning, judgment, and decision making. The first type of processes is generally assumed to be fast, automatic, and unconscious, whereas the second type is slow, effortful, and conscious. A concise overview and analysis of the premises and conclusions of these theories and a response to different arguments against dual-process models is given in [11] and [12]. It is important to mention that there is no evidence for one single generic dual-process model underlying all cognitive functions but merely that there is an empirical basis for a dual-process distinction when studying cognitive functions separately.

The dual-process modeling framework has been very successful in the fields of cognitive and social psychology; think about the influential research program on heuristics and biases of Kahneman and Tversky [13], the use of counting versus retrieval strategies in mental arithmetics [14–16], the study of the development of automaticity in cognitive tasks [17–19], or the automatic processing of social information in stereotyping [20]. In the field of psychometrics, however, dual-process models never really seem to have gained a foothold. The psychometrics literature has always been somewhat decoupled from that of cognitive psychology. Over the last 30 years, psychometrics and cognitive psychology have evolved separately into two research domains that rarely communicate. The majority of psychometric models deal with one particular process governed by one (or more) latent variables that leads to an observed response behavior. Our findings show that there is a clear need for reconciliation between psychometric and cognitive modeling. A similar conclusion was reached in [4]. Their two-level branching model, which we also use in this paper, is a prototypical example of a psychometric latent trait model that is partly inspired by cognitive modeling and that makes a clear distinction between ‘fast’ and ‘slow’ intelligence. This idea, once established, can have far-reaching consequences for psychometrics and encourages a closer collaboration between the fields of psychometrics and cognitive psychology.

## Methods

### Ethics statement

Participants, either their parents or their schools, agreed to the use of the anonymized data for scientific research when they subscribed to the Math Garden or Language Sea systems. The research described in this paper is approved by the ethics committee of the Faculty of Social and Behavioral Sciences of the University of Amsterdam (Lab Ethics Review Board). Project number: 2016-PML-6535.

### The Math Garden computerized adaptive practice framework

Math Garden [1] is a computerized adaptive learning environment in which children can practice their mathematical abilities. The Math Garden environment comprises over 20 different domains in which children can practice a specific mathematical skill. Domains range from basic arithmetic, such as addition or multiplication, to more involved tasks, such as the Set game or other IQ tasks. Each domain comprises several hundreds of items of varying difficulty. Most of the domains that are dealt with in this paper consist of open-ended items. The addition and subtraction domain consist of multiple choice items with 6 alternatives. Children can log in to the system and select a domain in which they want to practice. A game consists of 15 different items of the selected domain. For each item, a time limit of 20 seconds is imposed and the item is scored with the so-called Signed Residual Time (SRT) scoring rule. This scoring rule was introduced in [3] and has the following form:

$$\begin{array}{c} S_{i}=\left(2x_{i}-1\right)\left(1-t_{i}\right)\;, \end{array}$$ (9)

where *S*_*i*_ denotes the score earned after answering item *i* and *x*_*i*_ ∈ {1, 0} and *t*_*i*_ ∈ [0, 1] denote the accuracy and the response time (with the time limit scaled to 1), respectively. Instead of responding to the item, people also have the possibility to use the ‘question mark button’, in which case they earn a score of zero. The particular form of the scoring rule discourages guessing and imposes an explicit speed-accuracy trade-off. After every administration, the person’s ability (i.e., the ability corresponding to the selected domain) and the item’s difficulty are updated via an Elo rating algorithm [1]. Items are selected by the system such that the probability to answer an item correctly is about 0.75. Every administration (person, item, accuracy, response time, ratings, date) is saved in a database.

In our item pair study, which is summarized in Tables 3 and 4 and from which Table 1 describes a particular example item pair, we focus on a large collection of item pairs in the period between 01-03-2012 and 01-07-2014. For each item pair, we look for people who answered both items on the same day and extract their respective accuracies and response times from the Math Garden database, which are then used to build the contingency tables. We restrict ourselves to these people because a person’s ability can change substantially over time. By restricting ourselves to people who answered the item pair in one day, the assumption that their ability is fixed remains (approximately) valid. Responses that were given after the deadline or that were produced using the question mark button are not taken into account. The response *0* is not taken into account for multiplication items because this response was set as the default response for these items for some time. If an item is answered multiple times by the same person during the same day, only the first administration is used. In all studied contingency tables all observed response pairs are given by different persons.

### Restrictions imposed on item pair contingency tables by the two-level branching model and its truncations

We will derive the restrictions imposed on item pair contingency tables by the two-level branching model which is defined in Eq (7) and Fig 1. It is not hard to see that the joint distribution for *x*_*i*_ and *y*_*i*_ has the following form:

$$\begin{array}{c} P\left(x_{i},y_{i}|\theta ,b_{i}\right)=\frac{{\left(\frac{1+\text{exp}\xi_{i}^{\left(3\right)}}{1+\text{exp}\xi_{i}^{\left(2\right)}}\right)}^{y_{i}}\text{exp}\left(y_{i}\xi_{i}^{\left(1\right)}+x_{i}y_{i}\xi_{i}^{\left(2\right)}+x_{i}\left(1-y_{i}\right)\xi_{i}^{\left(3\right)}\right)}{\left(1+\text{exp}\xi_{i}^{\left(1\right)}\right)\left(1+\text{exp}\xi_{i}^{\left(3\right)}\right)}\;, \end{array}$$ (10)

where boldface symbols denote vectors in the index *s* ∈ {1, 2, 3}, where *s* refers to one of the nodes of the tree displayed in Fig 1. Consider a pair of items *i* and *j* with difficulties ***b***_*i*_ and ***b***_*j*_. According to the two-level branching model, the probability *P*(*x*_*i*_, *y*_*i*_, *x*_*j*_, *y*_*j*_|***θ***) to respond (*x*_*i*_, *y*_*i*_) and (*x*_*j*_, *y*_*j*_) conditional on ability ***θ***, is given by the following expression:

$$\begin{array}{c} P\left(x_{i},y_{i},x_{j},y_{j}|\theta \right)=P\left(x_{i},y_{i}|\theta ,b_{i}\right)P\left(x_{j},y_{j}|\theta ,b_{j}\right)\;, \end{array}$$ (11)

where *P*(*x*_*i*_, *y*_*i*_|***θ***, **b**_*i*_) is defined in Eq (10). The probabilities Eq (11) are written out in Table 6, in which the following definition is used

$$\begin{array}{c} Z_{ij}^{\left(1s\right)\left(1s^{\prime }\right)}=\left(1+\text{exp}\left(\xi_{i}^{\left(1\right)}\right)\right)\left(1+\text{exp}\left(\xi_{i}^{\left(s\right)}\right)\right)\left(1+\text{exp}\left(\xi_{j}^{\left(1\right)}\right)\right)\left(1+\text{exp}\left(\xi_{j}^{\left(s^{\prime }\right)}\right)\right)\;. \end{array}$$ (12)

Remember that $\xi_{i}^{\left(s\right)}=\theta^{\left(s\right)}-b_{i}^{\left(s\right)}$. To compare these expressions with the empirical findings (of Table 1, for example), we have to integrate out the *θ*^(1)^, *θ*^(2)^ and *θ*^(3)^ parameters:

$$\begin{array}{c} P\left(x_{i},y_{i},x_{j},y_{j}\right)=\int P\left(x_{i},y_{i},x_{j},y_{j}|\theta \right)f\left(\theta \right)\text{d}\theta^{\left(1\right)}\text{d}\theta^{\left(2\right)}\text{d}\theta^{\left(3\right)}\;, \end{array}$$ (13)

where *f*(***θ***) is the (unknown) distribution of *θ*^(1)^, *θ*^(2)^, and *θ*^(3)^ in the population. By writing out this expression for all 16 response patterns in the contingency table it is easy to demonstrate that this model is saturated on the item pair contingency table: it has a parameter for every cell. We therefore look at two particular truncations of the model that are not saturated on the contingency table: the 2P&3I (*θ*^(2)^ = *θ*^(3)^) and 3P&2I ($b_{i}^{\left(2\right)}=b_{i}^{\left(3\right)}$) truncations.

**Table 6:** Two-process model probabilities conditional on the latent variables *θ*^(1)^, *θ*^(2)^ and *θ*^(3)^.

	(*x*_*i*_ = 0, *y*_*i*_ = 1)	(*x*_*i*_ = 0, *y*_*i*_ = 0)	(*x*_*i*_ = 1, *y*_*i*_ = 0)	(*x*_*i*_ = 1, *y*_*i*_ = 1)
(*x*_*j*_ = 0, *y*_*j*_ = 1)	$\frac{\text{exp}(\xi_{i}^{\left(1\right)}+\xi_{j}^{\left(1\right)})}{Z_{ij}^{\left(12)(12\right)}}$	$\frac{\text{exp}(\xi_{j}^{\left(1\right)})}{Z_{ij}^{\left(13)(12\right)}}$	$\frac{\text{exp}(\xi_{i}^{\left(3\right)}+\xi_{j}^{\left(1\right)})}{Z_{ij}^{\left(13)(12\right)}}$	$\frac{\text{exp}(\xi_{i}^{\left(1\right)}+\xi_{i}^{\left(2\right)}+\xi_{j}^{\left(1\right)})}{Z_{ij}^{\left(12)(12\right)}}$
(*x*_*j*_ = 0, *y*_*j*_ = 0)	$\frac{\text{exp}(\xi_{i}^{\left(1\right)})}{Z_{ij}^{\left(12)(13\right)}}$	$\frac{1}{Z_{ij}^{\left(13)(13\right)}}$	$\frac{\text{exp}(\xi_{i}^{\left(3\right)})}{Z_{ij}^{\left(13)(13\right)}}$	$\frac{\text{exp}(\xi_{i}^{\left(1\right)}+\xi_{i}^{\left(2\right)})}{Z_{ij}^{\left(12)(13\right)}}$
(*x*_*j*_ = 1, *y*_*j*_ = 0)	$\frac{\text{exp}(\xi_{i}^{\left(1\right)}+\xi_{j}^{\left(3\right)})}{Z_{ij}^{\left(12)(13\right)}}$	$\frac{\text{exp}(\xi_{j}^{\left(3\right)})}{Z_{ij}^{\left(13)(13\right)}}$	$\frac{\text{exp}(\xi_{i}^{\left(3\right)}+\xi_{j}^{\left(3\right)})}{Z_{ij}^{\left(13)(13\right)}}$	$\frac{\text{exp}(\xi_{i}^{\left(1\right)}+\xi_{i}^{\left(2\right)}+\xi_{j}^{\left(3\right)})}{Z_{ij}^{\left(12)(13\right)}}$
(*x*_*j*_ = 1, *y*_*j*_ = 1)	$\frac{\text{exp}(\xi_{i}^{\left(1\right)}+\xi_{j}^{\left(1\right)}+\xi_{j}^{\left(2\right)})}{Z_{ij}^{\left(12)(12\right)}}$	$\frac{\text{exp}(\xi_{j}^{\left(1\right)}+\xi_{j}^{\left(2\right)})}{Z_{ij}^{\left(13)(12\right)}}$	$\frac{\text{exp}(\xi_{i}^{\left(3\right)}+\xi_{j}^{\left(1\right)}+\xi_{j}^{\left(2\right)})}{Z_{ij}^{\left(13)(12\right)}}$	$\frac{\text{exp}(\xi_{i}^{\left(1\right)}+\xi_{i}^{\left(2\right)}+\xi_{j}^{\left(1\right)}+\xi_{j}^{\left(2\right)})}{Z_{ij}^{\left(12)(12\right)}}$

The objects $Z_{ij}^{\left(1s\right)\left(1s^{\prime }\right)}$ are defined in Eq (12).

The 2P&3I truncation leads to the parametrization of the contingency table probabilities as displayed in Table 7. As can be seen from this table, these probabilities are completely determined by 14 independent parameters:

- 12 ‘score’ parameters {*φ*^(1)^, …, *φ*^(12)^} of which only 11 are independent because of the constraint
  $$\begin{array}{c} \sum_{k=1}^{12}\varphi^{\left(k\right)}=1\;,\;\text{and} \end{array}$$ (14)
- 3 ‘item’ parameters *α*_1_, *α*_2_ and *α*_3_.

The 3P&2I truncation leads to the parametrization of the contingency table probabilities as displayed in Table 8. As can be seen from this table, these probabilities are completely determined by 14 independent parameters:

- 14 ‘score’ parameters {*φ*^(1)^, …, *φ*^(14)^} of which only 13 are independent because of the constraint
  $$\begin{array}{c} \sum_{k=1}^{14}\varphi^{\left(k\right)}=1\;,\;\text{and} \end{array}$$ (15)
- 1 ‘item’ parameter *α*.

**Table 7:** Manifest 2P&3I probabilities.

	(*x*_*i*_ = 0, *y*_*i*_ = 1)	(*x*_*i*_ = 0, *y*_*i*_ = 0)	(*x*_*i*_ = 1, *y*_*i*_ = 0)	(*x*_*i*_ = 1, *y*_*i*_ = 1)
(*x*_*j*_ = 0, *y*_*j*_ = 1)	*φ*^(1)^	*φ*^(7)^	$\varphi^{\left(8\right)}\frac{1}{1+\alpha_{3}}$	$\varphi^{\left(2\right)}\frac{1}{1+\alpha_{1}}$
(*x*_*j*_ = 0, *y*_*j*_ = 0)	*φ*^(10)^	*φ*^(4)^	$\varphi^{\left(5\right)}\frac{1}{1+\alpha_{2}}$	$\varphi^{\left(11\right)}\frac{\alpha_{3}}{\alpha_{1}\alpha_{2}+\alpha_{3}}$
(*x*_*j*_ = 1, *y*_*j*_ = 0)	$\varphi^{\left(11\right)}\frac{\alpha_{1}\alpha_{2}}{\alpha_{1}\alpha_{2}+\alpha_{3}}$	$\varphi^{\left(5\right)}\frac{\alpha_{2}}{1+\alpha_{2}}$	*φ*^(6)^	*φ*^(12)^
(*x*_*j*_ = 1, *y*_*j*_ = 1)	$\varphi^{\left(2\right)}\frac{\alpha_{1}}{1+\alpha_{1}}$	$\varphi^{\left(8\right)}\frac{\alpha_{3}}{1+\alpha_{3}}$	*φ*^(9)^	*φ*^(3)^

Parametrization of the contingency table as implied by the the 2P&3I truncation of the two-level branching model. The parameters *φ*^(*k*)^ are subject to the constraint Eq (14).

**Table 8:** Manifest 3P&2I probabilities.

	(*x*_*i*_ = 0, *y*_*i*_ = 1)	(*x*_*i*_ = 0, *y*_*i*_ = 0)	(*x*_*i*_ = 1, *y*_*i*_ = 0)	(*x*_*i*_ = 1, *y*_*i*_ = 1)
(*x*_*j*_ = 0, *y*_*j*_ = 1)	*φ*^(1)^	*φ*^(7)^	*φ*^(8)^	$\varphi^{\left(2\right)}\frac{1}{1+\alpha }$
(*x*_*j*_ = 0, *y*_*j*_ = 0)	*φ*^(11)^	*φ*^(4)^	$\varphi^{\left(5\right)}\frac{1}{1+\alpha }$	*φ*^(12)^
(*x*_*j*_ = 1, *y*_*j*_ = 0)	*φ*^(13)^	$\varphi^{\left(5\right)}\frac{\alpha }{1+\alpha }$	*φ*^(6)^	*φ*^(14)^
(*x*_*j*_ = 1, *y*_*j*_ = 1)	$\varphi^{\left(2\right)}\frac{\alpha }{1+\alpha }$	*φ*^(9)^	*φ*^(10)^	*φ*^(3)^

Parametrization of the contingency table as implied by the the 3P&2I truncation of the two-level branching model. The parameters *φ*^(*k*)^ are subject to the constraint Eq (15).

#### Model estimation

It is not very hard to compute maximum likelihood estimates of the parameters of the 2P&3I and 3P&2I truncations on an item-pair contingency table. For both truncations it is straightforward to determine the score parameters *φ*^(*k*)^ because they are equal to the proportion of responses in the corresponding cells. For example, for the 2P&3I truncation *φ*^(8)^ can be estimated by computing the proportion of the observations in cells 3 and 14 (for the cell enumeration, see Table 1) over the total number of observations and for the 3P&2I truncation *φ*^(8)^ can be estimated by computing the proportion of the observations in cell 3 over the total number of observations. For the 2P&3I truncation, the maximum likelihood estimates for *α*_1_, *α*_2_ and *α*_3_ can be determined by solving the following system of maximum likelihood equations:

$$\begin{array}{ccc} \frac{n_{9}+n_{13}}{\alpha_{1}}-\frac{n_{4}+n_{13}}{1+\alpha_{1}}-\frac{\left(n_{8}+n_{9}\right)\alpha_{2}}{\alpha_{1}\alpha_{2}+\alpha_{3}} & = & 0\;,\\ \frac{n_{9}+n_{10}}{\alpha_{2}}-\frac{n_{7}+n_{10}}{1+\alpha_{2}}-\frac{\left(n_{8}+n_{9}\right)\alpha_{1}}{\alpha_{1}\alpha_{2}+\alpha_{3}} & = & 0\;,\\ \frac{n_{8}+n_{14}}{\alpha_{3}}-\frac{n_{3}+n_{14}}{1+\alpha_{3}}-\frac{n_{8}+n_{9}}{\alpha_{1}\alpha_{2}+\alpha_{3}} & = & 0\;, \end{array}$$ (16)

where the *n*_*k*_ indicate the number of observations in cell *k*. For the 3P&2I truncation, the maximum likelihood estimate for *α* is given by the following expression:

$$\begin{array}{c} \alpha =\frac{n_{10}+n_{13}}{n_{4}+n_{7}}\;. \end{array}$$ (17)

### Restrictions imposed on item pair contingency tables by a CIM

A generic CIM has the following expression for the joint probability distribution of *x*_*i*_ and *y*_*i*_:

$$\begin{array}{c} P\left(x_{i},y_{i}|\theta ,b_{i}\right)=\frac{\text{exp}\left(x_{i}\xi_{i}^{\left(1\right)}+y_{i}\xi_{i}^{\left(2\right)}\right)}{1+\text{exp}\xi_{i}^{\left(1\right)}+\text{exp}\xi_{i}^{\left(2\right)}+\text{exp}\left(\xi_{i}^{\left(1\right)}+\xi_{i}^{\left(2\right)}\right)}\;, \end{array}$$ (18)

where

$$\begin{array}{c} \xi_{i}^{\left(s\right)}=\theta^{\left(s\right)}-b_{i}^{\left(s\right)}\;, \end{array}$$ (19)

with *s* ∈ {1, 2}. Boldface symbols denote vectors in the *s* index, that is ***θ*** = (*θ*^(1)^, *θ*^(2)^) and $b_{i}=\left(b_{i}^{\left(1\right)},b_{i}^{\left(2\right)}\right)$. The first type of parameters $\left(\theta^{\left(1\right)},b_{i}^{\left(1\right)}\right)$ govern the accuracy, whereas the second type of parameters $\left(\theta^{\left(2\right)},b_{i}^{\left(2\right)}\right)$ govern the response time. Correlations between accuracy and response time are introduced by a second-level model *f*(***θ***) that governs the correlations between *θ*^(1)^ and *θ*^(2)^.

Let us derive the restrictions imposed by this model on item pair contingency tables. Consider a pair of items *i* and *j* with difficulties ***b***_*i*_ and ***b***_*j*_. According to the CIM, the probability *P*(*x*_*i*_, *y*_*i*_, *x*_*j*_, *y*_*j*_|***θ***) to respond (*x*_*i*_, *y*_*i*_) and (*x*_*j*_, *y*_*j*_) conditional on ability ***θ***, is given by the following expression:

$$\begin{array}{c} P\left(x_{i},y_{i},x_{j},y_{j}|\theta \right)=P\left(x_{i},y_{i}|\theta ,b_{i}\right)P\left(x_{j},y_{j}|\theta ,b_{j}\right)\;, \end{array}$$ (20)

where *P*(*x*_*i*_, *y*_*i*_|***θ***, ***b***_*i*_) is defined in Eq (18). To compare these expressions with the empirical findings (of Table 1, for example), we have to integrate out the *θ*^(1)^ and *θ*^(2)^ parameters:

$$\begin{array}{c} P\left(x_{i},y_{i},x_{j},y_{j}\right)=\int P\left(x_{i},y_{i},x_{j},y_{j}|\theta \right)f\left(\theta \right)\text{d}\theta^{\left(1\right)}\text{d}\theta^{\left(2\right)}\;, \end{array}$$ (21)

where *f*(***θ***) is the (unknown) distribution of *θ*^(1)^ and *θ*^(2)^ in the population. By writing out this expression for all 16 response patterns in the contingency table it is easy to demonstrate that this model leads to the parametrization of the contingency table probabilities as displayed in Table 9. As can be seen from this table, these probabilities are completely determined by 10 independent parameters:

- 9 ‘score’ parameters {*φ*^(1)^, …, *φ*^(9)^} of which only 8 are independent because of the constraint
  $$\begin{array}{c} \sum_{k=1}^{9}\varphi^{\left(k\right)}=1\;,\;\text{and} \end{array}$$ (22)
- 2 ‘item’ parameters *α*_1_ and *α*_2_.

**Table 9:** Manifest CIM probabilities.

	(*x*_*i*_ = 0, *y*_*i*_ = 1)	(*x*_*i*_ = 0, *y*_*i*_ = 0)	(*x*_*i*_ = 1, *y*_*i*_ = 0)	(*x*_*i*_ = 1, *y*_*i*_ = 1)
(*x*_*j*_ = 0, *y*_*j*_ = 1)	*φ*^(1)^	$\varphi^{\left(2\right)}\frac{1}{1+\alpha_{2}}$	$\varphi^{\left(4\right)}\frac{\alpha_{1}}{1+\alpha_{1}+\alpha_{2}+\alpha_{1}\alpha_{2}}$	$\varphi^{\left(6\right)}\frac{\alpha_{1}}{1+\alpha_{1}}$
(*x*_*j*_ = 0, *y*_*j*_ = 0)	$\varphi^{\left(2\right)}\frac{\alpha_{2}}{1+\alpha_{2}}$	*φ*^(3)^	$\varphi^{\left(5\right)}\frac{\alpha_{1}}{1+\alpha_{1}}$	$\varphi^{\left(4\right)}\frac{\alpha_{1}\alpha_{2}}{1+\alpha_{1}+\alpha_{2}+\alpha_{1}\alpha_{2}}$
(*x*_*j*_ = 1, *y*_*j*_ = 0)	$\varphi^{\left(4\right)}\frac{\alpha_{2}}{1+\alpha_{1}+\alpha_{2}+\alpha_{1}\alpha_{2}}$	$\varphi^{\left(5\right)}\frac{1}{1+\alpha_{1}}$	*φ*^(7)^	$\varphi^{\left(8\right)}\frac{\alpha_{2}}{1+\alpha_{2}}$
(*x*_*j*_ = 1, *y*_*j*_ = 1)	$\varphi^{\left(6\right)}\frac{1}{1+\alpha_{1}}$	$\varphi^{\left(4\right)}\frac{1}{1+\alpha_{1}+\alpha_{2}+\alpha_{1}\alpha_{2}}$	$\varphi^{\left(8\right)}\frac{1}{1+\alpha_{2}}$	*φ*^(9)^

Parametrization of the contingency table as implied by the CIM Eq (21). The parameters *φ*^(*k*)^ are subject to the constraint Eq (22).

#### Model estimation

It is not very hard to compute maximum likelihood estimates of the parameters on an item-pair contingency table. It is straightforward to determine the score parameters *φ*^(*k*)^ because they are equal to the proportion of responses in the corresponding cells. For example *φ*^(2)^ can be estimated by computing the proportion of the observations in cells 2 and 5 (for the cell enumeration, see Table 1) over the total number of observations and *φ*^(4)^ can be estimated by computing the proportion of the observations in cells 3, 8, 9 and 14 over the total number of observations. The maximum likelihood estimates for the *α*_1_ and *α*_2_ parameters can be determined by solving the following pair of maximum likelihood equations:

$$\begin{array}{ccc} \varphi^{\left(5\right)}\frac{\alpha_{1}}{1+\alpha_{1}}+\varphi^{\left(4\right)}\frac{\alpha_{1}+\alpha_{1}\alpha_{2}}{1+\alpha_{1}+\alpha_{2}+\alpha_{1}\alpha_{2}}+\varphi^{\left(6\right)}\frac{\alpha_{1}}{1+\alpha_{1}}+\varphi^{\left(7\right)}+\varphi^{\left(8\right)}+\varphi^{\left(9\right)} & = & O(x_{i})\;,\\ \varphi^{\left(2\right)}\frac{\alpha_{2}}{1+\alpha_{2}}+\varphi^{\left(4\right)}\frac{\alpha_{2}+\alpha_{1}\alpha_{2}}{1+\alpha_{1}+\alpha_{2}+\alpha_{1}\alpha_{2}}+\varphi^{\left(8\right)}\frac{\alpha_{2}}{1+\alpha_{2}}+\varphi^{\left(1\right)}+\varphi^{\left(6\right)}+\varphi^{\left(9\right)} & = & O(y_{i})\;, \end{array}$$ (23)

where *O*(*x*_*i*_) and *O*(*y*_*i*_) denote, respectively, the observed mean value of *x*_*i*_ and *y*_*i*_.

### Restrictions imposed on item pair contingency tables by a CDM

The SRT model of [3] is the prototypical example of a CDM. The joint probability distribution of *x*_*i*_ and *y*_*i*_ in the discretized (because the time variable is discrete) SRT model is given by

$$\begin{array}{c} P\left(x_{i},y_{i}|\theta ,b_{i}\right)=\frac{\text{exp}\left({\tilde{S}}_{i}\xi_{i}\right)}{\left(1+\text{exp}\left(\xi_{i}\right)\right)(1+\text{exp}\left(\xi_{i}/2\right))}\;, \end{array}$$ (24)

where

$$\begin{array}{c} \xi_{i}=\theta -b_{i}\; \end{array}$$ (25)

and the collapsed SRT score ${\tilde{S}}_{i}$ is defined as

$$\begin{array}{c} {\tilde{S}}_{i}=x_{i}y_{i}+\frac{1}{2}\left(x_{i}-y_{i}\right)+\frac{1}{2}\;. \end{array}$$ (26)

Notice that ${\tilde{S}}_{i}$ contains an explicit coupling of accuracy and response time *x*_*i*_ *y*_*i*_, which makes the model a CDM.

Let us derive the restrictions imposed by this model on item pair contingency tables. Consider a pair of items *i* and *j* with difficulties *b*_*i*_ and *b*_*j*_. According to the discretized SRT model, the probability *P*(*x*_*i*_, *y*_*i*_, *x*_*j*_, *y*_*j*_|*θ*) to respond (*x*_*i*_, *y*_*i*_) and (*x*_*j*_, *y*_*j*_) conditional on ability *θ*, is given by the following equation:

$$\begin{array}{c} P\left(x_{i},y_{i},x_{j},y_{j}|\theta \right)=P\left(x_{i},y_{i}|\theta ,b_{i}\right)P\left(x_{j},y_{j}|\theta ,b_{j}\right)\;, \end{array}$$ (27)

where *P*(*x*_*i*_, *y*_*i*_|*θ*, *b*_*i*_) is defined in Eq (24). To compare these probabilities with the empirical findings, we have to integrate out the *θ* parameter:

$$\begin{array}{c} P\left(x_{i},y_{i},x_{j},y_{j}\right)=\int P\left(x_{i},y_{i},x_{j},y_{j}|\theta \right)f\left(\theta \right)\text{d}\theta \;, \end{array}$$ (28)

where *f*(*θ*) is the distribution of *θ* in the population. By writing out this expression for all 16 response patterns in the contingency table it is easy to demonstrate that this model leads to the parametrization of the contingency table probabilities as displayed in Table 10. As can be seen from this table, these probabilities are completely determined by 7 independent parameters:

- 7 ‘score’ parameters {*φ*^(1)^, …, *φ*^(7)^} of which only 6 are independent because of the constraint
  $$\begin{array}{c} \sum_{k=1}^{7}\varphi^{\left(k\right)}=1\;\;\text{and} \end{array}$$ (29)
- 1 ‘item’ parameter *α*.

**Table 10:** Manifest CDM probabilities.

	(*x*_*i*_ = 0, *y*_*i*_ = 1)	(*x*_*i*_ = 0, *y*_*i*_ = 0)	(*x*_*i*_ = 1, *y*_*i*_ = 0)	(*x*_*i*_ = 1, *y*_*i*_ = 1)
(*x*_*j*_ = 0, *y*_*j*_ = 1)	*φ*^(1)^	$\varphi^{\left(2\right)}\frac{\alpha }{1+\alpha }$	$\varphi^{\left(3\right)}\frac{\alpha^{2}}{1+\alpha +\alpha^{2}}$	$\varphi^{\left(4\right)}\frac{\alpha^{3}}{1+\alpha +\alpha^{2}+\alpha^{3}}$
(*x*_*j*_ = 0, *y*_*j*_ = 0)	$\varphi^{\left(2\right)}\frac{1}{1+\alpha }$	$\varphi^{\left(3\right)}\frac{\alpha }{1+\alpha +\alpha^{2}}$	$\varphi^{\left(4\right)}\frac{\alpha^{2}}{1+\alpha +\alpha^{2}+\alpha^{3}}$	$\varphi^{\left(5\right)}\frac{\alpha^{2}}{1+\alpha +\alpha^{2}}$
(*x*_*j*_ = 1, *y*_*j*_ = 0)	$\varphi^{\left(3\right)}\frac{1}{1+\alpha +\alpha^{2}}$	$\varphi^{\left(4\right)}\frac{\alpha }{1+\alpha +\alpha^{2}+\alpha^{3}}$	$\varphi^{\left(5\right)}\frac{\alpha }{1+\alpha +\alpha^{2}}$	$\varphi^{\left(6\right)}\frac{\alpha }{1+\alpha }$
(*x*_*j*_ = 1, *y*_*j*_ = 1)	$\varphi^{\left(4\right)}\frac{1}{1+\alpha +\alpha^{2}+\alpha^{3}}$	$\varphi^{\left(5\right)}\frac{1}{1+\alpha +\alpha^{2}}$	$\varphi^{\left(6\right)}\frac{1}{1+\alpha }$	*φ*^(7)^

Discretized SRT model probabilities Eq (28) written out for all possible answer patterns. The score parameters *φ*^(*k*)^ are subject to the constraint Eq (29).

#### Model estimation

It is not very hard to compute maximum likelihood estimates of the parameters on an item-pair contingency table. It is straightforward to determine the score parameters *φ*^(*k*)^ because they are equal to the proportion of responses in the corresponding cells. For example *φ*^(2)^ can be estimated by computing the proportion of the observations in cells 2 and 5 (for the cell enumeration, see Table 1) over the total number of observations and *φ*^(4)^ can be estimated by computing the proportion of the observations in cells 4, 7, 10 and 13 over the total number of observations. The maximum likelihood estimate for the *α* parameter can be determined by solving the following maximum likelihood equation:

$$\begin{array}{ccc} & & \varphi^{\left(2\right)}\frac{\alpha /2}{1+\alpha }+\varphi^{\left(3\right)}\frac{\alpha /2+\alpha^{2}}{1+\alpha +\alpha^{2}}+\varphi^{\left(4\right)}\frac{\alpha /2+\alpha^{2}+3\alpha^{3}/2}{1+\alpha +\alpha^{2}+\alpha^{3}}+\\ & & \varphi^{\left(5\right)}\frac{1/2+\alpha +3\alpha^{2}/2}{1+\alpha +\alpha^{2}}+\varphi^{\left(6\right)}\frac{1+3\alpha /2}{1+\alpha }+\varphi^{\left(7\right)}\pi_{3}=O\left({\tilde{S}}_{i}\right)\;, \end{array}$$ (30)

where $O({\tilde{S}}_{i})$ denotes the observed mean value of ${\tilde{S}}_{i}$.
